# Mucosal-associated invariant T cells are a profibrogenic immune cell population in the liver

**DOI:** 10.1038/s41467-018-04450-y

**Published:** 2018-06-01

**Authors:** Pushpa Hegde, Emmanuel Weiss, Valérie Paradis, Jinghong Wan, Morgane Mabire, Sukriti Sukriti, Pierre-Emmanuel Rautou, Miguel Albuquerque, Olivia Picq, Abhishak Chandra Gupta, Gladys Ferrere, Hélène Gilgenkrantz, Badr Kiaf, Amine Toubal, Lucie Beaudoin, Philippe Lettéron, Richard Moreau, Agnès Lehuen, Sophie Lotersztajn

**Affiliations:** 1 Inserm UMR-1149, Centre de Recherche sur l’Inflammation, 75018 Paris, France; 2 Sorbonne Paris Cité, Laboratoire d’Excellence Inflamex, Faculté de Médecine, Site Xavier Bichat, Université Paris Diderot, 75018 Paris, France; 3 Département d’Anesthésie et Réanimation, Hôpital Beaujon, Assistance Publique-Hôpitaux de Paris, 92110 Clichy, France; 4 Département de Pathologie, Hôpital Beaujon, Assistance Publique-Hôpitaux de Paris, 92110 Clichy, France; 5 Département Hospitalo-Universitaire (DHU) UNITY, Service d’Hépatologie, Hôpital Beaujon, Assistance Publique-Hôpitaux de Paris, 92110 Clichy, France; 6 Inserm U-1016, CNRS UMR 8104, Institut Cochin, Université Paris-Descartes, 75014 Paris, France

## Abstract

Liver fibrosis is the common response to chronic liver injury, and leads to cirrhosis and its complications. Persistent inflammation is a driving force of liver fibrosis progression. Mucosal-associated invariant T (MAIT) cells are non-conventional T cells that display altered functions during chronic inflammatory diseases. Here, we show that circulating MAIT cells are reduced in patients with alcoholic or non-alcoholic fatty liver disease-related cirrhosis while they accumulate in liver fibrotic septa. Using two models of chronic liver injury, we demonstrate that MAIT cell-enriched mice show increased liver fibrosis and accumulation of hepatic fibrogenic cells, whereas MAIT cell-deficient mice are resistant. Co-culture experiments indicate that MAIT cells enhance the proinflammatory properties of monocyte-derived macrophages, and promote mitogenic and proinflammatory functions of fibrogenic cells, via distinct mechanisms. Our results highlight the profibrogenic functions of MAIT cells and suggest that targeting MAIT cells may constitute an attractive antifibrogenic strategy during chronic liver injury.

## Abstract (web summary)

Hepatic fibrosis represents the liver response to chronic injury and can lead to cirrhosis. Here the authors show that mucosal-associated invariant T cells mediate chronic inflammation and fibrogenesis in the liver by inducing a proinflammatory phenotype in macrophages and myofibroblasts and proliferation of the latter.

## Introduction

Hepatic fibrosis, the common response to chronic liver injury, ultimately leads to cirrhosis, a major public health problem worldwide^1,2^. In western countries, the prevailing causes of fibrosis and cirrhosis include chronic alcohol consumption and non-alcoholic fatty liver disease associated with obesity and type-2 diabetes^3,4^. Cirrhosis lacks definitive treatment, and liver transplantation is considered as the only option for end-stage liver disease. Extracellular matrix accumulation during chronic liver injury is driven by a heterogeneous population of myofibroblasts that migrate and accumulate at the site of injury^1,2,5^. Advances in the understanding of liver fibrosis pathogenesis have underscored the critical sustained inflammation originating from resident and infiltrating immune cells, that drives the fibrogenic process during liver injury via direct effects on fibrogenic cell proinflammatory and profibrogenic functions, but also contributes to its resolution^1,2,6^. In recent years, monocytes/macrophages and conventional T-cell subsets have received the most interest, but much less is known about the contribution and functions of non-conventional T-cell subsets in the fibrogenic process, in particular regarding the possible impact of innate-like lymphoid cells^7^.

Mucosal-associated invariant T (MAIT) cells are non-conventional T cells that express an evolutionarily conserved semi-invariant T cell antigen receptor (TCR) repertoire (made of an invariant Vα7.2-Jα33 in humans and Vα19-Jα33 in mice) and are restricted by the non-classical MHC-related molecule 1 (MR1)^8^. They are abundant in human blood, gut, and liver, and secrete cytokines such as IL-17, granzyme B (Gr-B), IFN-γ, and TNF. In healthy individuals, MAIT cells play a defensive role against pathogens, by preserving epithelial and mucosal layer integrity, and protecting against bacterial invasion and viral infections, in particular in the liver^8–15^. A pathogenic role in inflammatory diseases has also recently emerged, with consistent data reporting altered MAIT cell-functions during acute and chronic inflammatory injury, including obesity, diabetes, arthritis, or inflammatory bowel diseases^15–19^. In the present study, we assessed whether MAIT cells contribute to the pathogenesis of liver fibrosis. We show that MAIT cells display proinflammatory and profibrogenic functions during chronic liver injury. Our data unravel this non-conventional T-cell subset as a promising target for antifibrogenic therapy.

## Results

### Blood MAIT cells are altered in cirrhotic patients

**Fig. 1:**
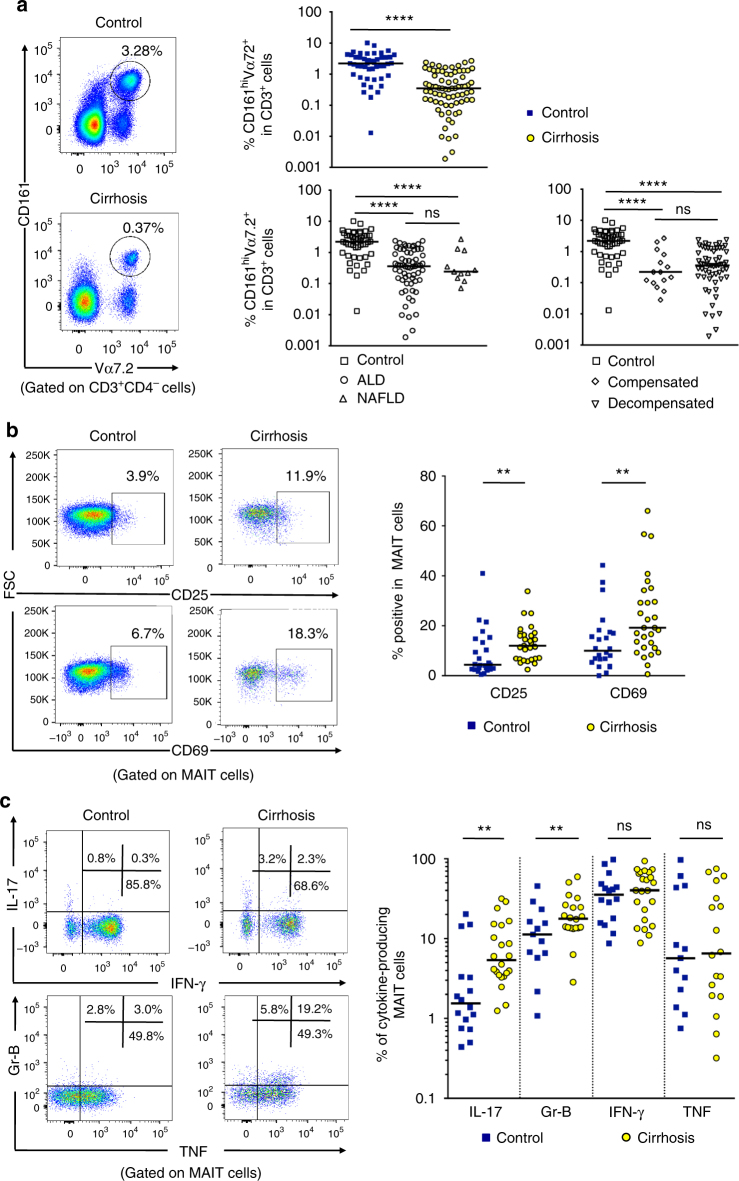
Frequency and functions of circulating MAIT cells are impaired during cirrhosis. **a** Representative dot plots showing reduction of CD161^hi^ Vα7.2^+^ double positive (MAIT) cells in PBMC and summary data from cirrhotic patients (*n* = 74), as compared to that in healthy donors (*n* = 47), and repartition of cirrhotic patients into disease etiology (alcoholic cirrhosis, *n* = 63 and NASH cirrhosis, *n* = 11) and severity (compensated cirrhosis, *n* = 15 and decompensated cirrhosis, *n* = 59). Representative dot plots and cumulative data of **b** increased surface expression of CD25 and CD69 on MAIT cells from healthy donors (*n* = 25) and cirrhotic patients (*n* = 29) and **c** cytokine profile of cirrhotic (*n* = 20–23) vs. healthy (*n* = 13–16) blood MAIT cells. Statistical analysis was performed using Mann–Whitney (**a**, **b**, **c**) or Kruskal–Wallis followed by Dunn’s post test analysis (**a**). **p ≤ 0.01; ****p ≤ 0.0001; ns *p* > 0.05

We first evaluated the frequency of circulating T-cell subsets in the peripheral blood mononuclear cells (PBMC) from severe (decompensated) and less severe (compensated) cirrhotic patients with alcoholic (ALD *n* = 63), and non-alcoholic fatty liver disease (NAFLD *n* = 11), and compared to that of healthy donors (control, *n* = 47) (see Supplementary Table 1, for clinical characteristics of the groups). There was a decrease in CD8^+^ T cells and a slight but significant increase in the CD4^+^ T-cell population in patients with cirrhosis. Detailed analysis of innate-like T-cell populations showed a small decrease in the frequency of iNKT cells in patients with cirrhosis, and no change in γδT cells (Supplementary Fig. 1a). However, as compared to healthy donors, the median MAIT cell frequency, identified as CD3^+^CD4^−^CD161^hi^ Vα7.2^+^ cells within the CD3^+^ population, was strongly decreased in patients with cirrhosis as compared to control (2.62% ± 0.3 in controls, within the range reported in other studies^16,20^ vs. 0.61% ± 0.07% in patients with cirrhosis, Fig. 1a). We also investigated whether clinical parameters may have an impact on blood MAIT cell frequency, in particular clinical complications of cirrhosis (i.e., compensated vs. decompensated), cirrhosis etiology (i.e., ALD vs. NAFLD) (Fig. 1a), or liver disease complications such as refractory ascites or encephalopathy (Table 1a). We found no significant association of either parameter on MAIT cell frequency. In addition, we did not find significant association of gender with MAIT cell frequency (Table 1a). It should be noted that the median age of the controls was significantly lower than that of patients (34 (29–53) vs. 57 (50–63) years), and that decreased MAIT cell frequency was significantly associated with age (Table 1a). However, using a bivariate analysis adjusted on age, we found that cirrhosis was still an independent predictor of lower blood MAIT cell frequency (Table 1b).

**Table 1:** Clinical characteristics associated with blood MAIT cell frequency using linear regression

Variable	Regression coefficient	95% CI		*p*
		Lower bound	Higher bound	
a. Univariate analysis				
Among the entire population				
Age	−0.034	−0.057	−0.011	<0.01
Male sex	−0.538	−1.16	0.084	0.09
Cirrhosis	−1.918	−2.426	−1.41	<0.01
Among patients with cirrhosis				
Alcohol-related cirrhosis	−0.187	−0.63	0.256	0.40
NASH-related cirrhosis	−0.04	−0.502	0.421	0.86
Child–Pugh score	0.004	−0.057	0.064	0.90
MELD score	0.001	−0.021	0.024	0.89
Ascites	0.019	−0.331	0.369	0.91
Encephalopathy	0.188	−0.217	0.594	0.36
Chronic antibiotic prophylaxis (norfloxacin and/or rifaximin) (%)	0.582	0.093	1.072	0.02
Beta-blockers	−0.016	−0.08	0.049	0.63
b. Bivariate analysis				
Among the entire population				
Age	−0.034	−0.057	−0.011	<0.01
Cirrhosis	−1.52	−2.206	−0.834	<0.01
Among patients with cirrhosis				
Age	0.012	−0.003	0.028	0.11
Chronic antibiotic prophylaxis (norfloxacin and/or rifaximin)	0.55	0.067	1.033	0.03

a. The potential relationship between patient characteristics and blood MAIT cell frequency was analyzed by linear regression univariate analysis

b. Each variable achieving a *p-*value < 0.05 was then introduced into a bivariate model, including the age to determine whether these variables predicted blood MAIT cell frequency independently of age

The majority of MAIT cells from control and cirrhotic patients were either CD8^+^ (nearly 80%) or double negative (CD8^−^CD4^−^, up to 20%). Blood MAIT cells from patients with cirrhosis displayed an activated phenotype, characterized by higher frequencies of CD25^+^ and CD69^+^ MAIT cells as compared to control individuals (Fig. 1b). Moreover, the mean fluorescence intensity of CD25 and CD69 on MAIT cells was negatively correlated with MAIT cell frequency (Supplementary Fig. 1b, c). MAIT cells from patients with cirrhosis produced more IL-17 and granzyme B than those from control individuals, whereas the frequency of IFN-γ^+^ or TNF^+^ MAIT cells was high, but not different between the two groups (Fig. 1c). Intriguingly, cirrhotic MAIT cells showed increased proliferation, as indicated by a higher frequency of Ki-67^+^ MAIT cells compared to control (Supplementary Fig. 1d). Moreover, the decrease in peripheral MAIT cell frequency was not related to activation-induced exhaustion, since there was no difference in the number of TIM-3^+^ and PD-1^+^ MAIT cells between the two groups (Supplementary Fig. 1e). The expression of Bcl-2 in MAIT cells was high both in control and cirrhotic patients (92.2% ± 2.9 vs. 89.5% ± 2.5), but showed no significant difference between groups. However, we found that the frequency of Bcl-2^+^ MAIT cells correlated to MAIT cell frequency (Supplementary Fig. 1f). These data suggest that the decrease in MAIT frequency may be related to decreased survival. Altogether, these data suggested that cirrhotic patients show a decreased frequency of blood MAIT cells, but with an activated phenotype.

We also analyzed whether preventive treatments usually administered to decompensated cirrhotic patients to avoid cirrhosis complications may modulate blood MAIT cell frequency, i.e., nonselective beta-blockers or long-term prophylactic antibiotic therapy (using norfloxacin or rifaximin that induce selective intestinal decontamination and prevent bacterial infections). We found no association with nonselective beta-blocker therapy (Table 1a). Strikingly, however, long-term prophylactic antibiotic therapy was significantly associated with a lower reduction in MAIT cell frequency (Table 1a, Supplementary Fig. 1g). Importantly, in bivariate analysis, the effect of long-term antibiotic therapy with norfloxacin or rifaximin was independent of age (Table 1b). We also found that antibiotic-exposed cirrhotic patients displayed significant reduction of CD25 expression (11.2 (5.2–12) vs. 14.5 (7.0–17.9), Supplementary Fig. 1h). Altogether, these data suggest that long-term antibiotic treatment partially prevents MAIT cell reduction and activation.

### Liver MAIT cells are altered in patients with cirrhosis

**Fig. 2:**
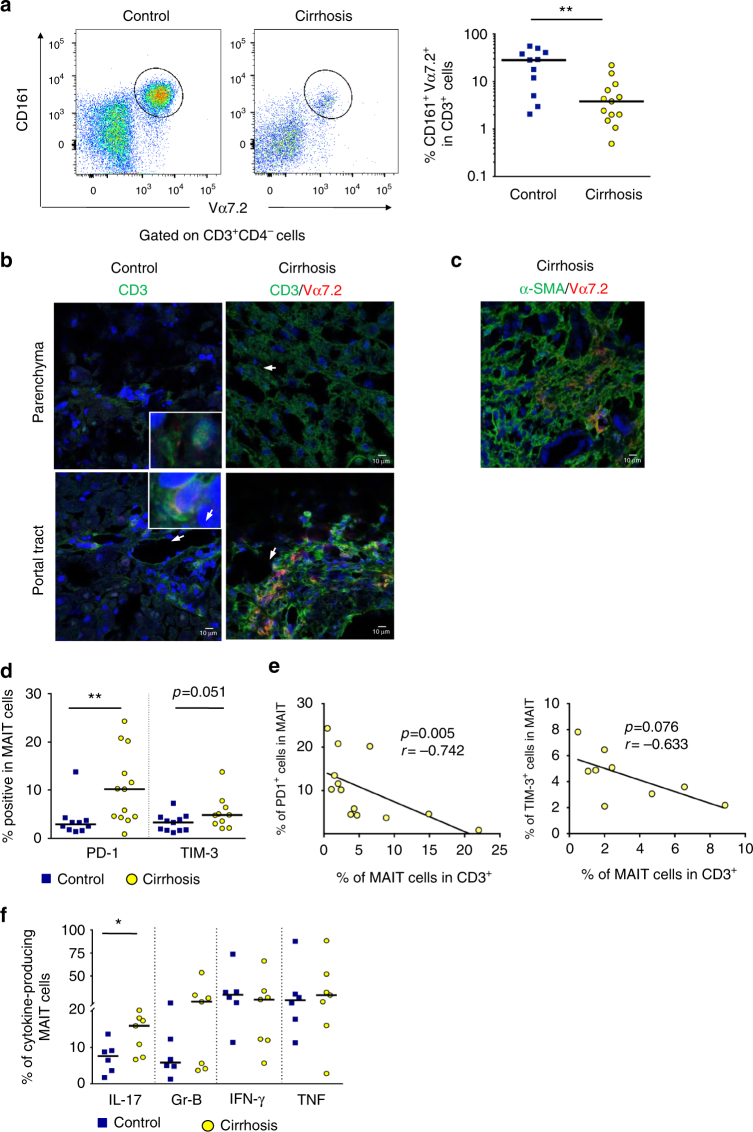
Frequency and functions of liver MAIT cells are impaired during cirrhosis. **a** Representative dot plots and summary data of CD161^hi^ Vα7.2^+^ double positive cells (MAIT) in mononuclear cells isolated from cirrhotic (*n* = 13) and control (*n* = 11) liver tissues. **b** Representative images of CD3^+^Vα7.2^+^ double positive cells in liver tissue sections from control (*n* = 4) and cirrhotic (*n* = 6) livers, showing the preferential presence of CD3^+^ Vα7.2^+^  cells within the fibrotic septa, and no or discrete sinusoidal immunostaining, whereas discrete sinusoidal staining is observed in control livers, both in sinusoidal space and portal tract (*n* = 6, double stained cells are indicated by arrows). Scale bar = 10 μm. **c** Representative CD3^+^ Vα7.2^+^ double immunostaining of liver tissue sections from cirrhotic livers, showing their presence close to α-SMA positive cells. Scale bar = 10 μm. **d** Comparison of expression of PD-1 and TIM-3 on intrahepatic MAIT cells in control (*n* = 10–11) and cirrhotic livers (*n* = 13–10). **e** Correlation between intrahepatic MAIT frequency and the percentage of positive MAIT cells for PD-1 (*n* = 13) and TIM-3 (*n* = 9) in cirrhotic livers. **f** Representative dot plots and summary data of cytokine profile of cirrhotic (*n* = 7) vs. control (*n* = 6) intrahepatic MAIT cells. Statistical analysis was performed using Mann–Whitney (**a**, **d**, **f**) or Spearman correlation test (**e**). *p ≤ 0.05; **p ≤ 0.01

We also studied the fate of MAIT cells in the cirrhotic liver, using isolated intrahepatic leukocytes (IHL) from liver explants of patients undergoing transplantation or resection. As expected^21^, the percentage of CD161^hi^ Vα7.2^+^ MAIT cells was higher in the liver than in the blood of control individuals (2.62% ± 0.3 in blood vs. 25.6% ± 5.7 in the liver). There was a decrease in the frequency of hepatic MAIT cells in cirrhotic livers as compared to control livers (Fig. 2a). Nevertheless, immunohistochemistry experiments revealed that, despite decreased frequency, hepatic distribution of Vα7.2^+^ cells showed striking differences between both groups as shown by confocal microscopy. Indeed, CD3^+^Vα7.2^+^ cells were present in the mesenchymal space within the fibrotic septa in cirrhotic livers, with discrete or even no staining in the sinusoids, whereas control livers showed exclusive CD3^+^Vα7.2^+^ immunoreactivity within the sinusoidal space (Fig. 2b, Supplementary Table 2). Interestingly, Vα7.2^+^ cells were found in proximity to fibrogenic myofibroblasts that are α-SMA positive cells^1^, mainly in the periportal area and along the fibrotic septa (Fig. 2c). MAIT cells displayed significant enhanced exhaustion features in cirrhotic livers, as reflected by an increased frequency of PD-1^+^ and TIM-3^+^ cells (Fig. 2d). In addition, there was a significant negative correlation between intrahepatic MAIT cell frequency and their expression of PD-1, and a tendency for TIM-3 (*p* = 0.07 by Spearman correlation test, Fig. 2e). There was a high frequency of CD25^+^ and CD69^+^ MAIT cells in control livers that was not further increased in cirrhotic livers (Supplementary Fig. 2a). These data suggest that expression of CD25 and CD69 might not be adequate to follow activation of MAIT cells in the liver, in line with reports showing that CD69 is also considered in non-lymphoid tissues as a retention molecule, highly expressed in a majority of resident memory T cells^22^. Finally, cirrhotic liver MAIT cells displayed an activated phenotype, characterized by higher frequencies of IL-17^+^ cells, but no difference in the number of MAIT cells producing granzyme B, IFN-γ, and TNF (Fig. 2f and Supplementary Fig. 2b).

Altogether, these data show major functional alterations in MAIT cell compartment during cirrhosis, with a strong decrease in blood MAIT cells associated with enhanced activation and exhaustion, and the presence of Vα7.2^+^ cells within the fibrotic septa in close contact with fibrogenic cells. We therefore investigated whether MAIT cells might alter the fibrogenic properties of hepatic myofibroblasts.

### MAIT cells enhance fibrogenic functions of myofibroblasts

**Fig. 3:**
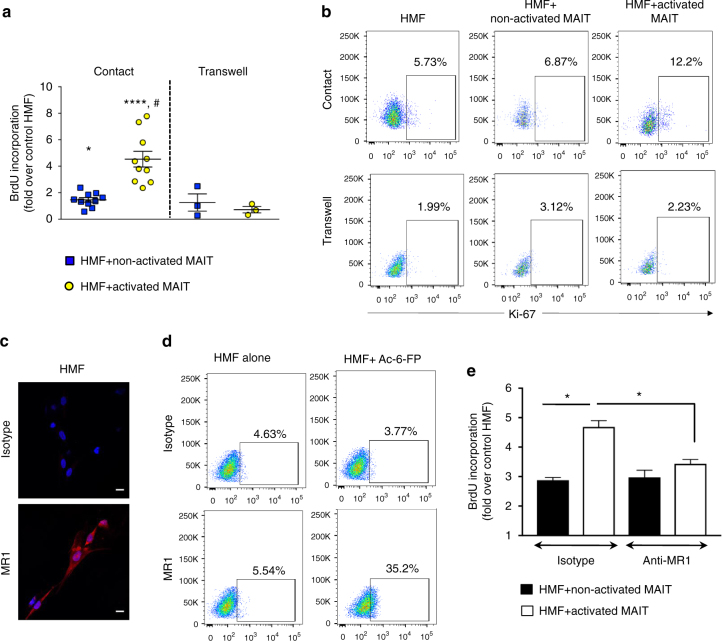
MAIT cells stimulate mitogenic functions of human hepatic myofibroblasts (HMF). **a** BrdU incorporation in hepatic myofibroblasts following direct and transwell co-cultures with either non-activated or activated MAIT cells. The results show the mean ± SEM of direct (*n* = 10) and transwell (*n* = 3) co-cultures, each performed with a different MAIT cell donor. **b** Representative dot plots showing the percentage of Ki-67^+^ hepatic myofibroblasts in direct and transwell co-culture with non-activated and activated MAIT cells by flow cytometry. **c** Representative immunofluorescence images showing expression of MR1 on hepatic myofibroblasts and no signal with control isotype. Similar results were obtained in three independent experiments. Scale bar = 10 μm. **d** Representative dot plots showing increased expression of MR1 on hepatic myofibroblast surface by flow cytometry, upon exposure to 1 μm Ac-6-FP. Three different cell cultures gave similar results. **e** DNA synthesis in hepatic myofibroblasts pretreated with MR1-neutralizing antibody or isotype, and co-cultured with either non-activated or activated MAIT cells. The results show a representative experiment and are the mean ± SEM of quadruplicate determinations. Similar results were obtained in three independent experiments with MAIT cells from three different donors (Supplementary Fig. 3b). Statistical analysis was determined using Mann–Whitney (**a**, **e**) or Kruskal–Wallis followed by Dunn’s post test analysis (**a**). *p ≤ 0.05; ****p ≤ 0.0001 vs. control HMF. #*p* ≤ 0.005 vs. HMF treated with non-activated MAIT cells

Hepatic myofibroblasts are fibrogenic cells that have a high proliferative capacity and accumulate at sites of liver injury in response to a wide variety of mitogens produced by neighboring cells^1,23^. We therefore assessed in co-culture experiments whether MAIT cells stimulate fibrogenic cell proliferation. Human hepatic fibrogenic cells in their fully activated myofibroblastic phenotype^24^, co-cultured with activated (anti-CD3/anti-CD28/IL-7-exposed) human MAIT cells, showed enhanced BrdU incorporation (Fig. 3a) or Ki-67 staining (Fig. 3b), while non-activated MAIT cells had a marginal but significant effect on the proliferative capacity of hepatic myofibroblasts (Fig. 3a, b). Moreover, activated MAIT cells significantly increased the proliferation of human hepatic myofibroblasts (HMF) compared to those co-cultured with non-activated MAIT cells. Surprisingly, there was no stimulation of hepatic myofibroblast DNA synthesis when transwell inserts separated MAIT cells from contact with hepatic myofibroblasts (Fig. 3a, b). These data suggested that direct contact rather than cytokine/chemokine production underlies MAIT cell-induced DNA synthesis of hepatic myofibroblasts. We therefore investigated whether TCR-dependent interaction via MR1 might transduce the mitogenic signal, since immunostaining and FACS analysis showed expression of MR1 by hepatic myofibroblasts (Fig. 3c, d). In addition, surface expression of MR1 on hepatic myofibroblasts was enhanced by acetyl-6-formyl-pterin (Ac-6-FP), a MR1 ligand that stabilizes MR1 at the plasma membrane^25^ (Fig. 3d). Functional mechanisms underlying MAIT cell-induced proliferation of hepatic myofibroblasts were investigated, using an MR1-neutralizing antibody. Strikingly, hepatic myofibroblast proliferation was significantly blunted in the presence of a MR1-neutralizing antibody as compared to isotype control (Fig. 3e and Supplementary Fig. 3b). Altogether, these data show that MAIT cells promote accumulation of fibrogenic cells by stimulating their proliferation in an MR1-dependent manner.

**Fig. 4:**
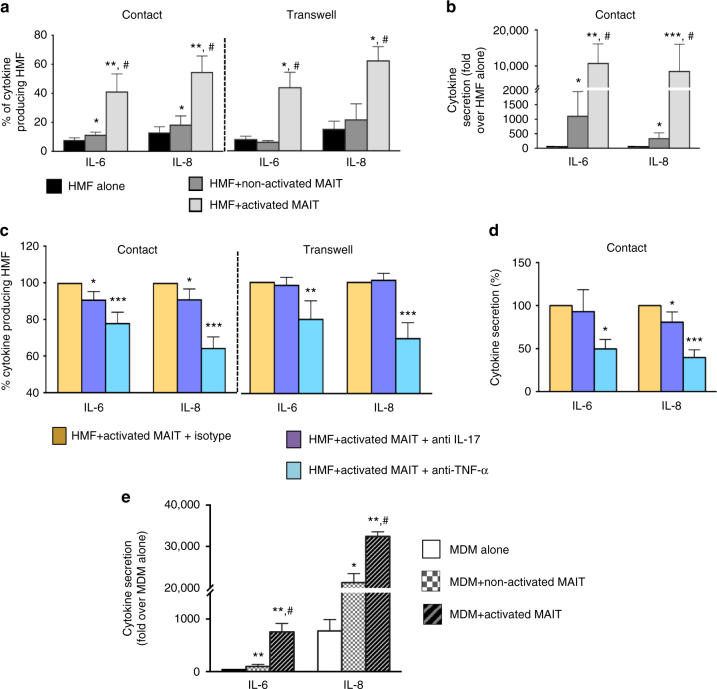
MAIT cells stimulate proinflammatory properties of human hepatic myofibroblasts. Analysis of intracellular cytokine production by human hepatic myofibroblasts **a** by FACS following direct (*n* = 8 experiments) or transwell (*n* = 6 experiments) co-cultured with either activated or non-activated MAIT cells; **b** by ELISA following direct co-cultures (*n* = 6 experiments). In each experiment, MAIT cells from different donors were used. **c**, **d** Analysis of intracellular cytokine production by human hepatic myofibroblasts co-cultured with activated or non-activated MAIT cells, following neutralization with 0.5 μg/ml of an IL-17-neutralizing antibody, 5 μg/ml of TNF-neutralizing antibody, or isotype. **c** FACS analysis (*n* = 6 experiments); **d** ELISA (*n* = 4 experiments). The results are expressed as the percentage of cytokine producing HMF in the presence of MAIT cells with neutralizing antibodies relative to those with isotype. **e** Analysis of IL-6 and IL-8 production by ELISA from monocyte-derived macrophages (MDM) upon direct co-culture with either activated or non-activated MAIT cells. The results show a representative experiment and are the mean ± SEM of sextuplicate determinations. Similar results were obtained in two independent co-culture experiments with both MAIT cells and MDM coming from two different donors (Supplementary Fig. 4b). Statistical significance was determined using Kruskal–Wallis followed by Dunn’s post test analysis (**a**–**e**). Comparison of HMF alone and HMF with non-activated and activated MAIT cells was performed further by Mann–Whitney test (**a**–**e**). *p ≤ 0.05; **p ≤ 0.01; ***p ≤ 0.001 vs. control HMF. #*p* ≤ 0.005 vs. HMF treated with non-activated MAIT cells

Hepatic myofibroblasts are also key contributors of the hepatic inflammatory response by producing proinflammatory mediators upon stimulation by various cytokines such as IL-17 and TNF^1,2,23,26–28^. Since activated MAIT cells are well characterized as IL-17 and TNF-producing cells^8^, we investigated whether MAIT cell-derived TNF or IL-17 secretion might affect the proinflammatory properties of hepatic myofibroblasts. In co-culture experiments, activated MAIT cells enhanced the production of IL-6 and IL-8 by hepatic myofibroblasts, as shown by FACS analysis and ELISA (Fig. 4a, b, Supplementary Fig. 4a); similar results were obtained in transwell experiments (Fig. 4a). These data suggested that MAIT cell-derived soluble mediators enhance the proinflammatory properties of hepatic myofibroblasts. FACS analysis and ELISA revealed that addition of a TNF-neutralizing antibody to the co-cultures or transwell chamber abrogated IL-6 and IL-8 production by hepatic myofibroblasts. In contrast, IL-17 blockade had a marginal effect (Fig. 4c, d). These findings demonstrate that TNF produced by MAIT cells promotes cytokine secretion by hepatic myofibroblasts.

These data unravel mitogenic and proinflammatory effects of MAIT cells on hepatic myofibroblasts via distinct pathways, involving direct MR1-mediated cell contact, and indirect TNF-mediated cell stimulation, respectively.

### MAIT cells stimulate cytokine release from macrophages

Release of proinflammatory cytokines by macrophages is a key feature in the pathogenesis of chronic liver diseases as it fosters an inflammatory environment that sustains the fibrogenic process. We therefore assessed whether MAIT cells may also interact with monocyte-derived macrophages. Co-cultures of activated MAIT cells with naive monocyte-derived macrophages stimulated the release of proinflammatory cytokines IL-6 and IL-8 (Fig. 4e and Supplementary Fig. 4b).

Collectively, these data demonstrate that MAIT cells trigger proinflammatory cytokine release by both hepatic myofibroblasts and macrophages.

### Profibrogenic properties of MAIT cells in vivo

**Fig. 5:**
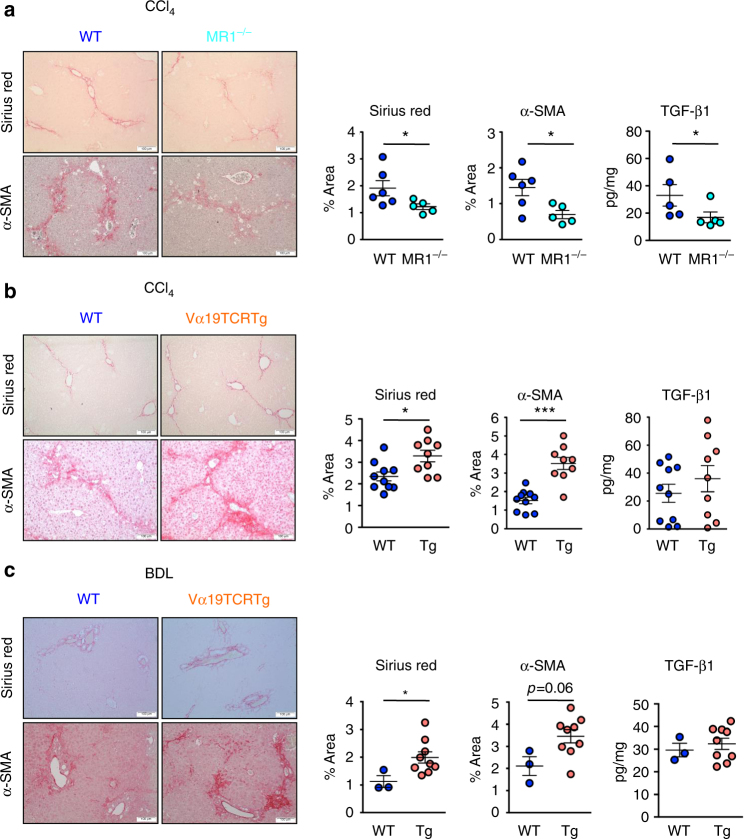
Profibrogenic properties of MAIT cells in vivo. Representative images and quantification of Sirius red and α-SMA areas, and hepatic TGF-β1 secretion in **a** MAIT cell-deficient (MR1^−/−^) mice (*n* = 5) and their WT littermates (*n* = 6) chronically administered with CCl_4_, **b** MAIT cell-enriched (Vα19TCRTg) mice (*n* = 9) and their WT littermates (*n* = 10) chronically exposed to CCl_4_ in two independent experiments (exp 1, *n* = 5 WT, *n* = 4 Tg; exp 2, *n* = 5 WT, *n* = 5 Tg), and **c** MAIT cell-enriched (Vα19TCRTg) mice (*n* = 9) and their WT littermates (*n* = 3) subjected to bile duct ligation. Scale bar = 100 μm. Statistical analysis was performed using Mann–Whitney (**a**, **b**, **c**). *p ≤ 0.05 vs. WT

Since Vα7.2^+^ cells were present within the fibrotic septa in the cirrhotic liver and MAIT cells enhanced the mitogenic and proinflammatory properties of hepatic myofibroblasts and promote the release of cytokines from macrophages, we next investigated the impact of MAIT cells on experimental liver fibrogenesis in vivo, taking advantage of the availability of MAIT cell-deficient mice (MR1^−/−^)^29^ and mice carrying a 10-fold increase in MAIT cell frequency (Vα19TCRTg)^30^. MR1^−/−^ or Vα19TCRTg mice showed no difference in CCl_4_-induced liver injury, as shown by the levels of serum alanine transaminase (Supplementary Fig. 5a) similar to that of WT littermates. However, CCl_4_-exposed MR1^−/−^ were resistant to fibrosis, as evidenced by decreased morphometry analysis of Sirius red staining, lower number of fibrogenic cells, as illustrated by reduced α-smooth muscle actin (α-SMA) staining, and reduced hepatic production of the profibrogenic cytokine TGF-β1 (Fig. 5a). Conversely, MAIT cell-enriched mice (Vα19TCRTg) exposed to CCl_4_ displayed exacerbated fibrosis as compared to WT counterparts, as reflected by enhanced Sirius red staining and increased number of α**-**SMA positive cells (Fig. 5b). In a pilot study, we found that similar increases in Sirius red staining and accumulation of α**-**SMA positive cells were observed in Vα19TCRTg transgenic animals undergoing bile duct ligation as compared to WT counterparts (Fig. 5c). However, there was no further increase in hepatic TGF-β production in Vα19TCRTg mice exposed to CCl_4_ or undergoing bile duct ligation (Fig. 5b, c) as compared to WT counterparts. In addition, we found no difference in the hepatic levels of TNF between WT animals and either MR1-deficient or Vα19TCRTg exposed to CCl_4_ or undergoing bile duct ligation (Supplementary Fig. 5b). Livers from mice lacking MAIT cells or enriched in MAIT cells exposed to CCl_4_ did not show differences in the frequency of CD4^+^ and CD8^+^ T lymphocytes, B lymphocytes, neutrophils, macrophages, and dendritic cells between the two groups in the different models, demonstrating that the resistance of MR1^−/−^ mice to fibrosis and the exacerbation in Vα19TCRTg were not secondary to changes in other immune cell populations (Supplementary Fig. 5c, d). Of note, as previously described in other tissues^17,31^, MR1^−/−^ mice harbored a similar frequency of iNKT cells and γδT cells in the liver as control littermates (Supplementary Fig. 5e).

Collectively, these data highlight the profibrogenic properties of MAIT cells in the liver.

## Discussion

Recent advances in the understanding of liver fibrosis pathogenesis have revealed that dysregulation of the immune system is a key factor leading to cirrhosis and liver failure, suggesting that manipulation of specific immune cell subsets may serve as the basis for antifibrotic strategies^1,2,5–7,32^. Combining human data in cirrhotic patients with cell culture experiments and in vivo models of fibrosis in MAIT cell-deficient or MAIT cell-enriched mice, the present study identifies activated MAIT cells as a novel major actor of the fibrogenic process.

Our data demonstrate that MAIT cell frequency is decreased in the blood of patients with chronic liver diseases. This reduction was observed both in patients with alcoholic and non-alcoholic fatty liver disease, and was similarly observed in patients with severe or less-severe cirrhosis. However, blood MAIT cells from patients with cirrhosis display an activated and proinflammatory profile, characterized by increased CD25 and CD69 expression and higher production of IL-17 and granzyme B. These findings are in line with data obtained in patients with viral (HIV, HCV, and HIV/HCV)^14,15,33,10,11^ or bacterial infections^8,9^, or patients with inflammatory diseases, including type-1 or type-2 diabetes, arthritic disease, inflammatory bowel disease, or systemic lupus erythematosus^16–19,34^. The fate of circulating MAIT cells in inflammatory diseases, and in particular whether they die or migrate to the target tissue, remains unclear in humans. Of note, we recently reported an increased migration of MAIT cells from the blood to the inflammed pancreas of NOD (non-obese diabetic) mice during disease progression toward diabetes^17^. Our results do not support increased exhaustion of circulating MAIT cells in cirrhotic patients, as we found no difference in the frequency of PD-1^+^ or TIM-3^+^ blood MAIT cells between control individuals and patients with cirrhosis. However, the frequency of circulating MAIT cells was significantly inversely correlated with their Bcl-2 expression, suggesting that the decreased frequency of MAIT cells in blood might be related to death by apoptosis following activation.

Interestingly, there was a significant association between MAIT cell frequency and age (Table 1), in keeping with recent reports in Scandinavian and British cohorts^35,36^. However, unlike Novak et al., who reported a gender difference of blood MAIT cells in fertile women vs. men of the same age^33^, we found no significant association of MAIT cell frequency with gender in our cohort. Nevertheless, a major finding of our study is that when adjusted on age, cirrhosis was still an independent predictor of lower blood MAIT cell frequency.

From a clinical perspective, identification of interventional treatments modulating MAIT cell frequency and/or phenotype is a major challenge. Interestingly, we found that long-term prophylactic antibiotic therapy (using norfloxacin or rifaximin) in patients with decompensated cirrhosis was associated with lower MAIT cell frequency reduction, and a decrease in their activation marker CD25. These data suggest that long-term prophylactic antibiotic therapy in severe cirrhotic patients impacts MAIT cell frequency and phenotype. However, further studies are needed to extend these results in a larger cohort and analyze the outcome of such treatment on disease progression.

Although the frequency of MAIT cells is much higher in the liver than in the blood, the frequency of liver MAIT cells was also reduced and expressed an exhausted status in patients with cirrhosis as compared to controls, in line with intrahepatic depletion of MAIT cells in patients with HCV infection^10^. However, and as observed in the blood, cirrhotic liver MAIT cells displayed a proinflammatory phenotype, characterized by a higher frequency of IL-17^+^-positive cells. Despite reduced MAIT cell frequency in the cirrhotic liver, immunohistochemistry experiments combining Vα7.2 and α-SMA immunostaining revealed that Vα7.2^+^ cells were systematically present in the mesenchymal space, within the fibrotic septa, in close contact to hepatic fibrogenic cells. Interestingly, a moderate number or no Vα7.2^+^ cells was found in the sinusoidal space of cirrhotic livers, whereas Vα7.2 staining in CD3^+^ cells was consistently observed in the sinusoidal space of control liver. These data suggest that the presence of activated MAIT cells in a fibrotic environment may result from their redistribution within the liver. These results are in line with data reported in patients with Crohn’s disease, in whom MAIT cells accumulate in the injured part of the ileum as compared to the normal ileum of the same patients^37^. Yet, these data emphasized MAIT cell-hepatic myofibroblast proximity and surface contact, providing the first argument that MAIT cells may interact with fibrogenic cells during chronic liver injury.

Various immune subsets play a critical role in the regulation of fibrosis progression and regression by controlling hepatic myofibroblast accumulation, including innate and adaptive immune cells such as monocytes/macrophages, dendritic cells, or B and T lymphocytes ^1,2,5–7,32^. Our findings unravel MAIT cells as a novel immune cell player with profibrogenic and proinflammatory properties. Indeed, MAIT cell-overexpressing mice show enhanced fibrosis in two experimental models, associated with an increase in the accumulation of α-SMA positive fibrogenic cells. As a mirror, mice deficient in MAIT cells are resistant to fibrosis and the density of fibrogenic cells is decreased. A critical feature of the fibrogenic process is the high mitogenic capacity of hepatic fibrogenic cells, leading to their accumulation in the fibrotic septa during chronic liver injury^1,2,5,6,32^. In line with in vivo experiments, a major finding of our study is that MAIT cells display mitogenic effects for hepatic myofibroblasts. Co-culture experiments show that the mitogenic properties of activated MAIT cells do not result from the release of mitogens for hepatic myofibroblasts, but rather from direct cell–cell contact, since the effect is lost in transwell experiments. It has been reported that human hepatic fibrogenic cells express a large number of MHC molecules, including the human leukocyte antigen members (HLA-I and HLA-II), but also lipid-presenting molecules (CD1b and CD1c)^38^. Concordantly, we identified the non-classical MHC-related molecule MR1 at the cell membrane of hepatic myofibroblasts, and found that hepatic myofibroblast DNA synthesis is decreased upon incubation of co-cultures with a neutralizing MR1 antibody. Although it has been extensively reported that proliferation of hepatic myofibroblasts is stimulated by a large variety of growth factors expressed during chronic liver injury^1,2,6,32^, our findings highlight that the mitogenic properties of MAIT cells rely on a novel mechanism, involving TCR/MR1-dependent interactions. Of note, MAIT cells stimulate hepatic myofibroblast proliferation without exogenous addition of MAIT ligand into the medium, suggesting that at least initially, MAIT cell–myofibroblast contact occurs in the absence of bacterial antigens. However, during chronic liver injury, increase in gut permeability, intestinal bacteria overgrowth, and dysbiosis are the characteristic features of patients with chronic liver diseases from various etiologies, or in experimental models of chronic liver injury, allowing gut bacteria to flow to the liver^3,4,39^. These data suggest that bacterial-derived MAIT ligands generated from vitamin B2 metabolites and host-derived methylglyoxal^40^ could accumulate in the liver during chronic liver injury. Nevertheless, enhanced accumulation of hepatic myofibroblasts following MR1-dependent contact with MAIT cells appears as a critical determinant of the profibrogenic functions of MAIT cells.

Another characteristic of hepatic fibrogenic cells is their proinflammatory properties^1^. Our findings demonstrate that activated MAIT cells promote a shift of hepatic myofibroblasts toward a proinflammatory phenotype, characterized by enhanced production of IL-8 and IL-6. The results of our co-culture experiments indicated that the proinflammatory functions of MAIT cells rely on a cytokine-dependent effect, since it was similarly induced in direct contact or transwell co-cultures. Several studies have conclusively demonstrated that TNF and IL-17 are major components of the fibrogenic and proinflammatory response during chronic liver injury^27,28^, suggesting that both cytokines may be likely candidates released by MAIT cells. Analysis of hepatic myofibroblast inflammatory profile showed abrogation of IL-8 and IL-6 production in the presence of a TNF-neutralizing antibody, whereas, surprisingly, IL-17 had no or a marginal effect, indicating that TNF, but not IL-17, is a main component of MAIT cell-mediated hepatic myofibroblast proinflammatory functions. It should nevertheless be noted that we found no difference in the hepatic TNF levels between WT, MR1^−/−^, and Vα19TCRTg mice exposed to CCl_4_ or undergoing bile duct ligation. However, TNF is not exclusively produced by MAIT cells, but can be released by activated macrophages or dendritic cells, and display profibrogenic functions by enhancing survival^28^ and proinflammatory^41^ functions of hepatic myofibroblasts. Therefore, variations in global hepatic TNF levels may not reflect local production of the cytokine by MAIT cells at the proximity of hepatic myofibroblasts. In addition, we cannot rule out that other mitogenic/proinflammatory mediators produced in low amounts by MAIT cells in culture experiments may contribute to MAIT cell-induced proinflammatory phenotype. Finally, we also provide evidence that MAIT cells contribute to liver inflammation by enhancing the release of inflammatory cytokines by monocyte-derived macrophages, which are key elements in the progression of the fibrogenic process. These data demonstrate that MAIT cells foster the inflammatory environment required for development of fibrosis and cirrhosis by promoting the release of inflammatory cytokines at least from hepatic myofibroblasts and macrophages.

In conclusion, these data extend our knowledge on the general properties of MAIT cells. They also add to our understanding of the mechanisms underlying inflammation-driven fibrogenesis and unravel this non-conventional T-cell subset as a promising target for antifibrogenic therapy.

## Methods

### Human blood samples

Blood samples were obtained with written informed consent from 74 patients with biopsy-proven cirrhosis, due to alcoholic (*n* = 63) or non-alcoholic fatty liver disease (*n* = 11) hospitalized at Hôpital Beaujon (Clichy, France) (Supplementary Table 1). Severity of cirrhosis was assessed using Child–Pugh score^42^. Child–Pugh grade-A cirrhosis was considered as compensated, whereas decompensated cirrhosis included Child–Pugh grades B and C. The study was approved by the local ethics committees (Comité d’Evaluation de l’Ethique des projets de Recherche Biomédicale (CEERB) Paris Nord: n°16–039). Blood from healthy volunteers control (*n* = 47) was obtained through a formalized agreement with French Blood Agency (Etablissement Français du Sang, agreement n° 2015012778). Blood was obtained by the agency after informed consent of the donors, in accordance with the Declaration of Helsinki. All experiments were approved by the INSERM Institutional Review Board and ethics committee. Data collection and analyses were performed anonymously. Non-inclusion criteria were evidence of recent gastrointestinal bleeding, current bacterial infections, and treatment with immunosuppressive drugs in the past 30 days and the presence of human immunodeficiency virus infection.

### PBMC isolation

PBMC were prepared from the blood using Ficoll-Hypaque (GE Healthcare) density gradient centrifugation, as previously described^43^, and freshly used for the analysis of surface phenotype of MAIT cells. For the detection of cytokine production, PBMC were stimulated for 6 h at 37 °C with PMA and ionomycin (Sigma-Aldrich) at 25 ng/ml and 1 μg/ml, respectively, in the presence of brefeldin A at 10 μg/ml (BioLegend) in RPMI medium supplemented with 10% fetal bovine serum (Life Technologies).

### Human liver samples

Cirrhotic liver samples (*n* = 13 cirrhotic and *n* = 11 controls for FACS analysis; *n* = 5 cirrhotic, *n* = 4 controls, and *n* = 3 livers with mild fibrosis (F1) for immunohistochemistry, respectively) were obtained from surgical samples (resection or liver transplantation) at a distance, when present from the tumor nodule. Normal or subnormal liver samples were obtained from patients who had no alteration in liver biological tests but underwent resection surgeries for non-hepatocellular primary tumor or colorectal cancer liver metastasis. Cirrhotic liver samples were obtained from either non-tumoral part of hepatocellular carcinoma resection or liver explant during liver transplantation. Liver tissue was processed as rapidly as possible after resection, frozen in liquid nitrogen, and stored at −80 °C (Biobank Pathology Dpt., Beaujon Hospital, DC-2009-938). For all cases, fibrosis staging (Supplementary Table 2) was assessed according to Metavir system^44^. All patients signed an informed consent form and the study was approved by the local Ethics Committee (Comité d’Evaluation de l’Ethique des projets de Recherche Biomédicale (CEERB) Paris Nord, IRB 00006477).

### Isolation of IHL from human liver

Explanted fresh liver tissue was shredded into 5-mm^3^ cubes in RPMI with 10% FCS. After mechanical dissociation of liver pieces, the single-cell suspension was depleted from hepatocytes and the non-parenchymal fraction was subjected to density gradient centrifugation using Ficoll Hypaque at 800 g for 20 min. The lymphocyte layer was collected, washed twice with RMPI, and used for the analysis of MAIT cell phenotype.

### Flow cytometric analysis

Flurochrome-conjugated anti-CD3, anti-CD4 (OKT4), anti-CD161 (HP-3G10), anti-Vα7.2^+^ (3C10), anti-CD25 (BC96), anti-CD69 (FN50), anti-CCR6 (G034E3), anti-Bcl-2 (clone 100), anti-IFN-γ (4S.B3), anti-granzyme B (GB11), anti-TNF (MAb11), anti-IL-17 (BL168), and anti-Ki-67 (B56) antibodies were obtained from BioLegend, France. Anti-CD8α (SK1) and anti-TCRβ™ (B1) antibodies were obtained from BD Biosciences. Dead cells were excluded from the analysis using the fixable viability dye eFlour 506 (eBiosciences). MAIT cells were identified by multicolor flow cytometry as CD3^+^CD4^–^CD8^+^CD161^hi^Vα7.2^+^ cells. Intracellular cytokines were analyzed using PMA/ionomycin/brefeldin A-treated PBMC using the intracellular cytokine staining kit (BD Biosciences), according to manufacturers’ instructions. Analysis of Ki-67 and Bcl-2 in PBMC was performed using the intracellular transcription factor staining kit (eBioscience). Data acquisition was performed using a BD Biosciences LSR Fortessa cytometer and data were analyzed using FlowJo analysis software (Tree Star) (gating strategy, Supplementary Fig. 6).

### Human MAIT cell isolation

MAIT cells were isolated from PBMC prepared from buffy coats of healthy donors, as previously described^16^. Briefly, monocytes and CD4 T cells were sequentially depleted from PBMC by adhesion on polystyrene culture flasks for 3 h at 37 °C, and anti-CD4 microbeads (Miltenyi Biotech, France), respectively. Vα7.2^+^ cells were isolated from the non-monocyte and non-CD4 PBMC fraction, using an anti-Vα7.2^+^ antibody conjugated to FITC, followed by a positive selection with anti-FITC microbeads (Miltenyi Biotech, France) and used as enriched MAIT cells for co-culture experiments. The purity of isolated Vα7.2^+^ cells was >80% and >95% of the isolated Vα7.2^+^ cells were co-expressing CD161. More than 95% of the isolated CD161^hi^ Vα7.2^+^ cells were positive to APC-conjugated 5-OP-RU-loaded MR1 tetramers^45^ (NIH Tetramer Core Facility, Emory University Vaccine Center, Atlanta, USA) (Supplementary Fig. 3). MAIT cells added in co-culture experiments were either left nonactivated or were activated using anti-CD3 (HIT3a) at 2.5 μg/ml, soluble anti-CD28 (CD28.2) (1 μg/ml), and IL-7 at 10 ng/ml (BioLegend)^21^.

### Co-culture experiments

HMFs were obtained by outgrowth of explants prepared from surgical specimen of normal human liver, as we previously described^46^. This procedure was performed in accordance with ethical regulations imposed by the French legislation. The fibrogenic phenotype of these cells has been extensively characterized^46^. During the first passage, MYCOKILL (Brunschwig Chemie B.V., the Netherlands) was included to the cultures. Then, cells were grown to 80% confluency in RPMI containing 10% fetal calf serum and serum deprived for 2 days before adding freshly isolated non-activated or activated MAIT cells, at the ratio of 1:10 (HMF:MAIT cells).

Monocyte-derived macrophages were obtained by differentiating human monocytes from healthy donors. Briefly, plastic-adhered monocytes from healthy donor PBMC were plated in RPMI with 10% FCS for 7 days. Suspended cells were washed off and the adhered macrophages were used for co-culture with non-activated and activated MAIT cells.

### DNA synthesis assay

BrdU (Roche, France) was added at the bottom of the well (transwell experiments), or to the co-culture medium for 18 h. In co-culture experiments, MAIT cells were then carefully aspirated, and adherent HMF were washed once with 1× PBS and their DNA synthesis was estimated by a colorimetric BrdU ELISA test (Roche, France) as per the manufacturer’s instruction. For the experiments with MR1-blocking antibodies, HMFs were incubated with purified anti-MR1 antibody at 20 μg/ml (26.5, BioLegend, France) or with isotype control antibody for 2 h at 37 °C. Pre-activated or non-activated MAIT cells were washed and co-cultured with anti-MR1 (26.5, BioLegend)-exposed HMF. Cells were then processed for DNA synthesis as described above. In separate experiments, FACS analysis of Ki-67 was performed in HMF upon co-culture with MAIT cells, using phycoerythrin (PE)-conjugated anti-Ki-67 (BioLegend, France) by intracellular staining as per the manufacturer’s instructions.

### Analysis of MR1 expression by immunocytochemistry

HMF were fixed in 4% paraformaldehyde, followed by incubation with a PBS-blocking buffer containing 1% BSA, 0.1% Triton X-100, and anti-MR1 antibody (clone 26.5, mouse IgG2a, kappa, 1:25, BioLegend) followed by goat anti-mouse IgG (H + L) secondary antibody, Alexa Fluor 555 (1:1000, Invitrogen). Nuclear staining was performed using Prolong Gold antifade mounting with DAPI (Invitrogen). No staining was observed with the isotype (clone MOPC-173, mouse IgG2a, kappa, 1:25, BioLegend). Cells were visualized using confocal microscopy (Confocal Zeiss LSM 780).

### Analysis of surface expression of MR1 by flow cytometry

Hepatic myofibroblast cultures or HMF/MAIT cell co-cultures were exposed to 1 μM acetyl-6-formyl-pterin (Ac-6-FP) (Schircks Laboratories, Switzerland) for 2 h at 37 °C. Cells were then washed, trypsinized, labeled with PE-conjugated anti-MR1 antibody (26.5)/isotype control, and subjected to flow cytometric analysis.

### Analysis of cytokine and chemokine production

Hepatic myofibroblasts were co-cultured with either non-activated or activated MAIT cells for 18 h. Brefeldin A (10 μg/ml) was added for the last 3 h, and cells were analyzed for intracellular cytokines and chemokines, using the intracellular cytokine staining kit (BD Biosciences). When indicated, IL-17- neutralizing antibody at 0.5 μg/ml (64CAP17, eBioscience), TNF-neutralizing antibody (Mab11, BioLegend) at 5 μg/ml, or isotype were added to the co-culture. Secreted levels of IL-8 and IL-6 were measured by ELISA (eBioscience), according to the manufacturer’s instruction.

### Animals

MR1^−/−^ C57BL/6J^29^, Vα19 Tg C57BL/6J mice^30^, and their WT counterparts were obtained from Olivier Lantz (Institut Curie, Paris, France), and bred in our animal facilities (agreement CEEA no. 34: 15–059 and C2EA 121 no. 02529.02). Since MR1 is the restriction molecule required for MAIT thymic development, MR1^−/−^ C57BL/6J do not have MAIT cells. On the contrary, mice expressing the Vα19-Jα33 TCR transgene have a 10-fold increase in MAIT cell frequency in the various tissues such as spleen, liver, colon, and lymph nodes. Both lines were further backcrossed onto C57BL/6J Jackson mice. Then two crosses were set up to generate on one hand MR1^−/−^ and MR1^−/+^ C57BL/6J littermates and on the other hand Vα19-Jα33 transgenic and their negative littermates. Mice were genotyped at 2 weeks of age and at weaning, mice were separated according to their genotype and kept in separated cages.

### Mice models of liver fibrosis

Animals were housed in pathogen-free animal facility and fed ad libitum. Liver fibrosis was induced in male mice (12–14-week old) by either repeated injections of carbon tetrachloride (CCl_4_, 0.5 ml/kg body weight, 1:10 dilution in mineral oil, Sigma, France), twice a week for 4 weeks (Vα19 TCR transgenic, *n* = 9; WT littermates *n* = 10 obtained from two independent experiments (exp 1, *n* = 5 WT, *n* = 4 Tg; exp 2, *n* = 5 WT, *n* = 5 Tg); MR1^−/−^, *n* = 5; WT littermates *n* = 6), or bile duct ligation and section (Vα19 TCR transgenic, *n* = 9; WT littermates *n* = 3), as we previously described^47,48^. Animals were sacrificed 24 h after the last CCl_4_ injection or 12 days after surgery in BDL mice. Experiments were performed in accordance with protocols approved by the Paris-Nord ethical committee C2EA 121 (authorization number 02529.02).

### Histological analysis

Sirius Red staining was performed on 4-µm-thick formalin-fixed paraffin-embedded tissue sections at the Pathology Department of Hôpital Bichat, Paris, France. Sirius Red-stained areas from ten fields (magnification 10×) from each mouse were quantified with ImageJ.

### Immunohistochemistry of mice liver

Immunohistochemical detection of α-SMA was carried out as previously described^47^ on paraffin-embedded mice liver tissue sections (4 μm) using the MOM immunodetection kit (Vector, PK2002) and a mouse monoclonal anti-α-SMA antibody (1:1000, Sigma, 2547) according to the manufacturer’s instructions. α-SMA positive area from ten fields (magnification 20×) from 3 to 7 mice/group were quantified with ImageJ. No staining was observed when the primary antibody was omitted.

### Cytokine analysis in liver homogenates

TGF-β1 and TNF levels were measured in the liver homogenates by ELISA (eBiosciences), according to the manufacturers’ instructions.

### Immunohistochemistry of human liver

Vα7.2 immunodetection was performed in frozen sections (10 μm) of normal (F0, *n* = 4) or subnormal (F1, *n* = 3) liver samples from patients who underwent resection surgeries for non-hepatocellular primary tumor (*n* = 3), colorectal (*n* = 2), or kidney (*n* = 1) cancer liver metastasis, and showed no (F0 *n* = 4) or mild (F1 *n* = 3) fibrosis and no alteration in liver biological tests. Liver samples from patients with cirrhosis were obtained from non-tumoral part of HCC resection (*n* = 3) or liver explant during liver transplantation (*n* = 2).

Tissue sections were incubated overnight at 4 °C with either anti-human CD3 (DAKO, 1:50 in PBS containing 1% BSA and 0.2% Triton X-100) or anti-human α-SMA (BioLegend, 1:200). Slides were then incubated for 1 h at room temperature with secondary antibody-HRP working solution, followed by Opal 570 fluorophore. Incubation with purified anti-human Vα7.2 antibody (BioLegend, 1:50) was performed overnight at 4 °C, followed by incubation in Alexa Fluor 488 goat anti-mouse IgG (1:200, Life Technologies) for 1 h at room temperature. Nuclear counterstaining was obtained with DAPI (1:10,000, Thermo). Images were acquired by confocal microscopy (Confocal Zeiss LSM 780). Vα7.2-positive cells were assessed (0: absent; +: positive) and their location was determined (sinusoidal space and mesenchymal space).

### Statistical analysis

The results are expressed as mean ± standard error of the mean (SEM) or median (interquartile range), as indicated. Mann–Whitney test was used to calculate significant levels between two groups. For comparison of means from multiple groups against one control group, Kruskal–Wallis with Dunn’s multiple comparison post test analysis was performed. Correlations were performed using nonparametric Spearman test. Fisher’s test was used to analyze the presence or absence of Vα7.2^+^ cells by immunohistochemistry in control and cirrhotic livers. All *p*-values are two-sided, and *p*-values less than 0.05 were considered to be statistically significant. The potential relationship between patient characteristics and blood MAIT cell frequency was analyzed by linear regression univariate analysis. Each variable achieving a *p-*value < 0.05 was then introduced into a bivariate model. Analyses were performed using GraphPad Prism version 6 and SPSS 22.0 (SPSS Inc., Chicago, IL, USA). Sample sizes were adequate to detect large effects between groups, as determined by the reproducibility and variability of each particular experiment and limited by the availability of patient and animal samples. No randomization or blinding was used.

### Data availability

The data that support the findings of this study are available from the corresponding author upon reasonable request.

## Electronic supplementary material

Supplementary Information
