# Supplementary material for: Mucosal-associated invariant T cells are a profibrogenic immune cell population in the liver
Source: Nat Commun. 2018 Jun 1;9:2146. doi: 10.1038/s41467-018-04450-y (PMC5984626; doi:10.1038/s41467-018-04450-y)
Supplement: Supplementary file 1 — Supplementary Information [file 41467_2018_4450_MOESM1_ESM.pdf]

Supplementary Information, Hegde et.al.

Mucosal-Associated invariant T cells are a profibrogenic immune cell population in the liver.

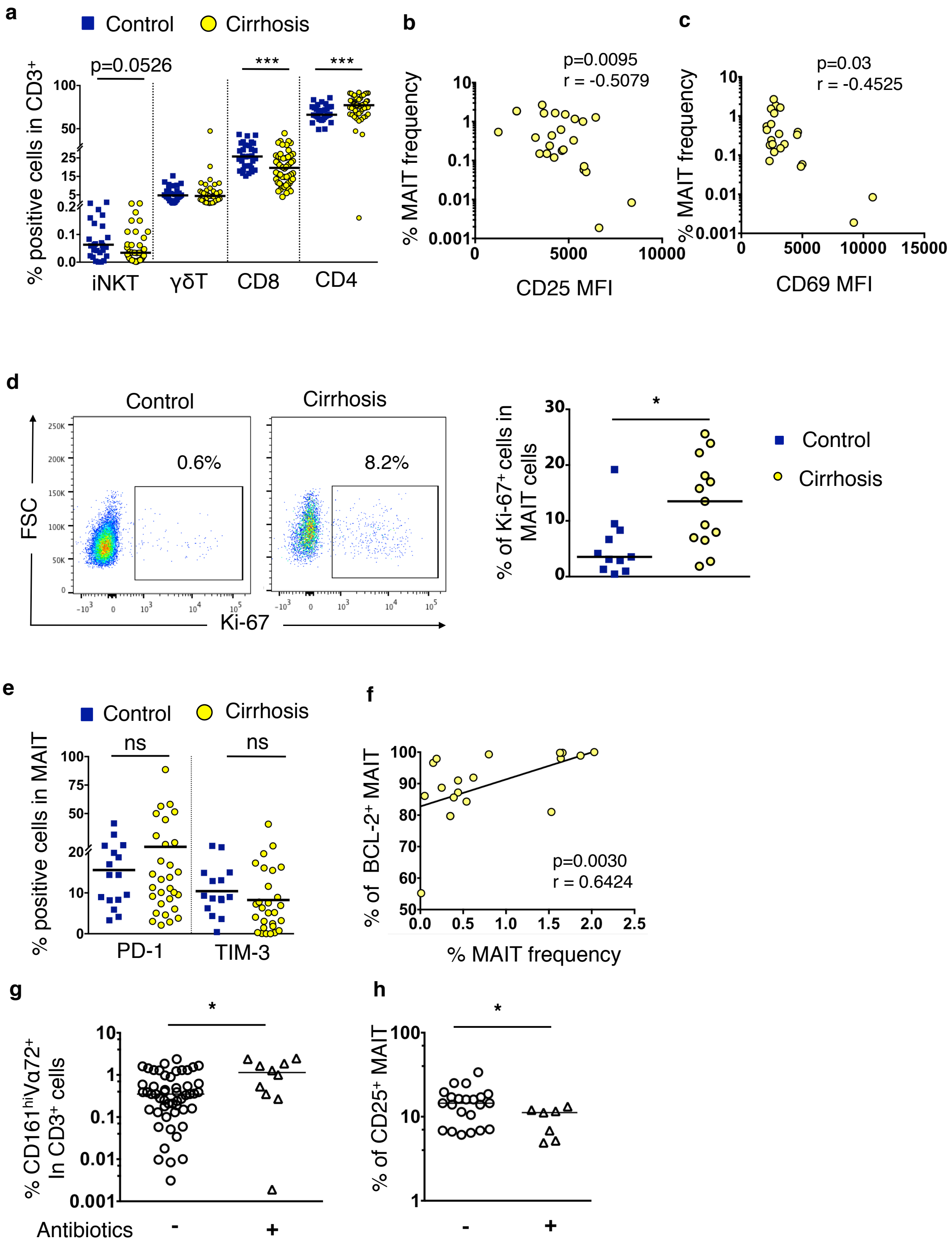

Supplementary Figure 1. (a) Comparison of circulating T lymphocyte subsets in patients with cirrhosis (n=41-54) and healthy donors (n=29-32). **(b and c)** Correlation of MAIT cell frequency with the mean fluorescence intensity (MFI) of CD25 (n=25) **(b)** or CD69 (n=22) **(c)**. **(d)** Representative histogram and cumulative results of Ki-67 expression in MAIT cells from cirrhotic patients (n=13) in comparison with healthy donors (n=11). **(e)** Comparison of expression of PD-1 and TIM-3 on blood MAIT cells in control (n=14-16) and cirrhotic livers (n=28-30). **(f)** Correlation of MAIT cell frequency with their BCL-2 expression in cirrhotic patients (n=14). **(g-h)** Comparison of **(g)** MAIT cell frequency **(h)** CD25 expression in cirrhotic patients with (n=10-7) or without (n=52-22) long-term prophylactic antibiotic therapy. Statistical analysis was performed using Mann-Whitney **(a, d, e, g, h)** or Spearman correlation test **(b, c, f)**. *$p \leq 0.05$; **$p \leq 0.01$; ***$p \leq 0.001$; ns $p > 0.05$.

a

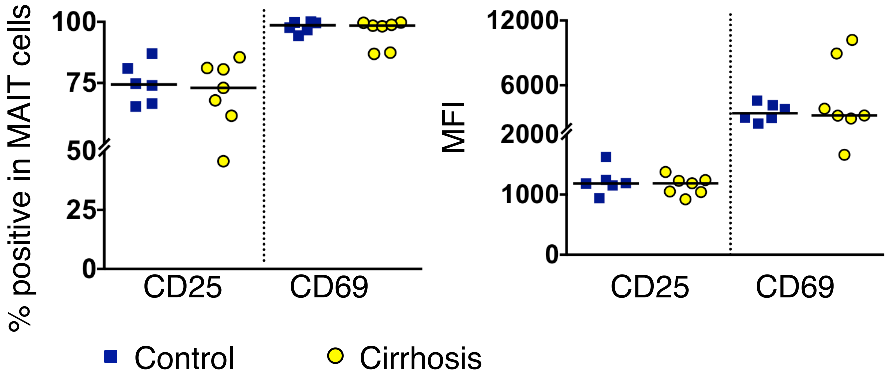

b

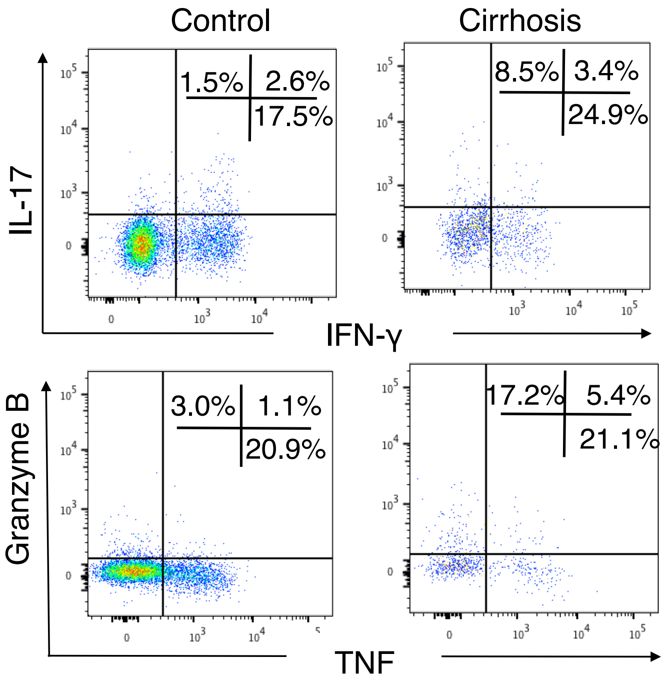

Supplementary Figure 2. (a) Summary data showing the surface expression of CD25 and CD69 on MAIT cells from cirrhotic (n=7) vs control (n=6) livers. **(b)** Representative dot plots and summary data of cytokine profile of cirrhotic (n=7) vs control (n=6) hepatic MAIT cells. Statistical analysis was determined using Mann-Whitney test **(a)**.

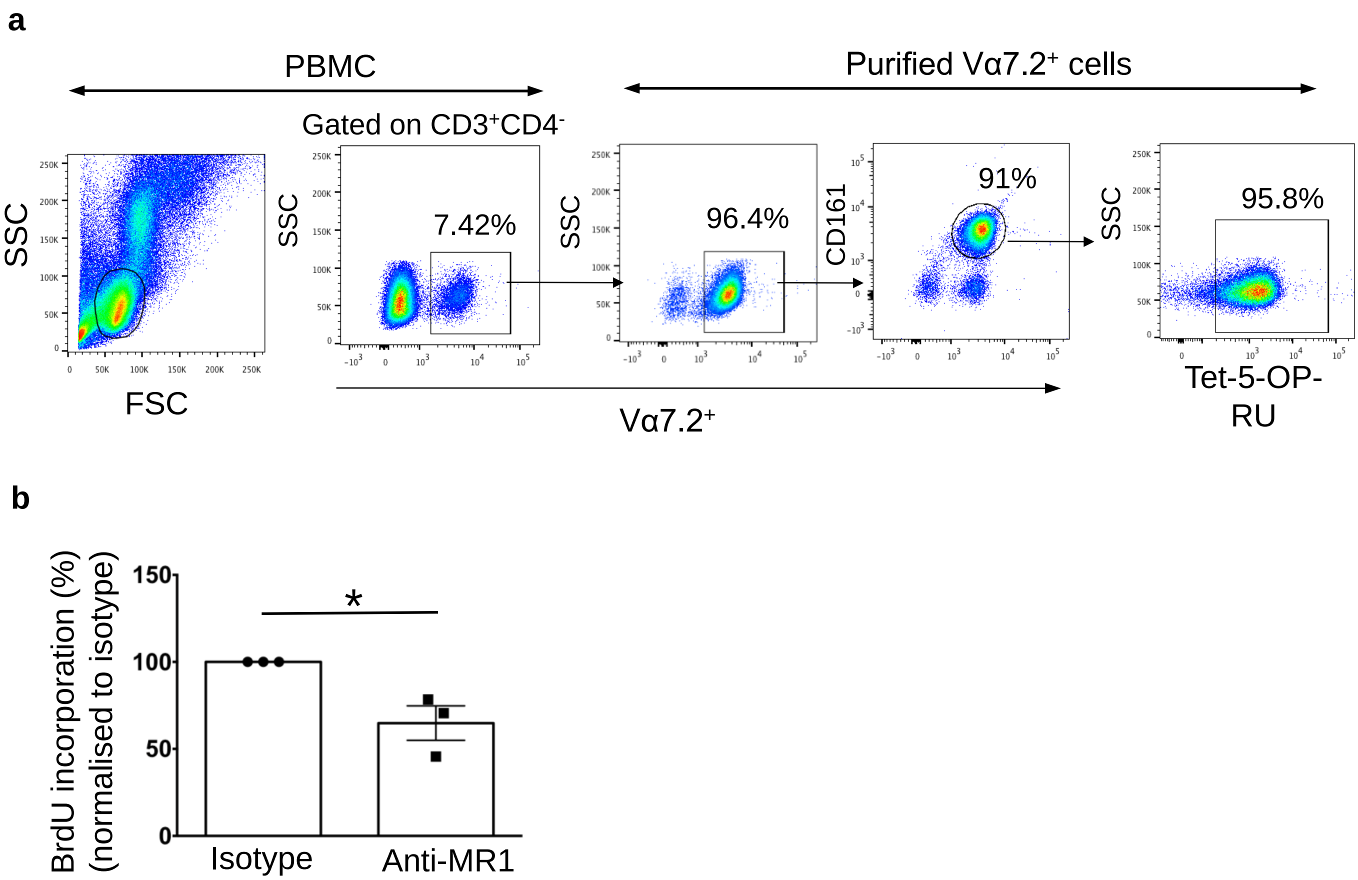

Supplementary Figure 3: (a) A typical dot plot showing isolated Vα7.2^{+} cells from healthy PBMC co-stained with CD161. More than 95% of the isolated MAIT cells were CD161^{+}Vα 7.2^{+} and also positive to 5-OP-RU loaded MR1. **(b)** DNA synthesis in hepatic myofibroblasts pre-treated with MR1 neutralizing antibody or isotype, and co-cultured with activated MAIT cells. Results show the mean±SEM of three independent experiments using three different donors of MAIT cells and are expressed as percent of BrdU incorporation in co-cultures of isotype-exposed HMF with activated MAIT cells. Statistical significance was analysed by unpaired t test. *$p \leq 0.05$.

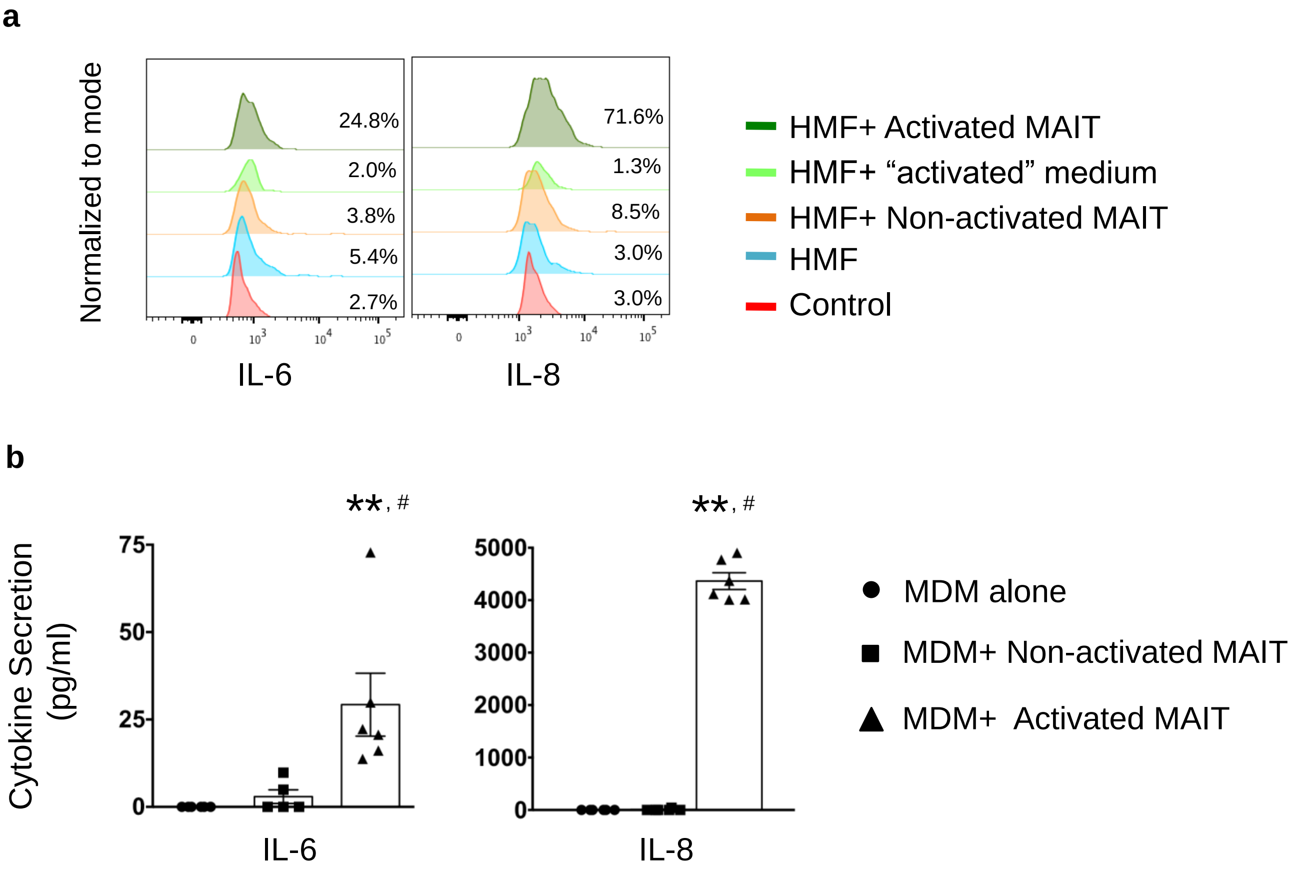

Supplementary Figure 4: **(a)** Representative dot plots of intracellular cytokine production by human hepatic myofibroblasts co-cultured with activated or non-activated MAIT cells, by FACS. **(b)** Analysis of IL-6 and IL-8 production by ELISA from monocyte-derived-macrophages (MDM) upon direct co-culture with either activated or non-activated MAIT cells. Results show a replicate experiment of Fig 4e with another MAIT cell and macrophage donor and are the mean±SEM of sextuplicate determinations. **$p \leq 0.01$; vs control MDM. #$p \leq 0.005$, vs MDM treated with non-activated MAIT cells.

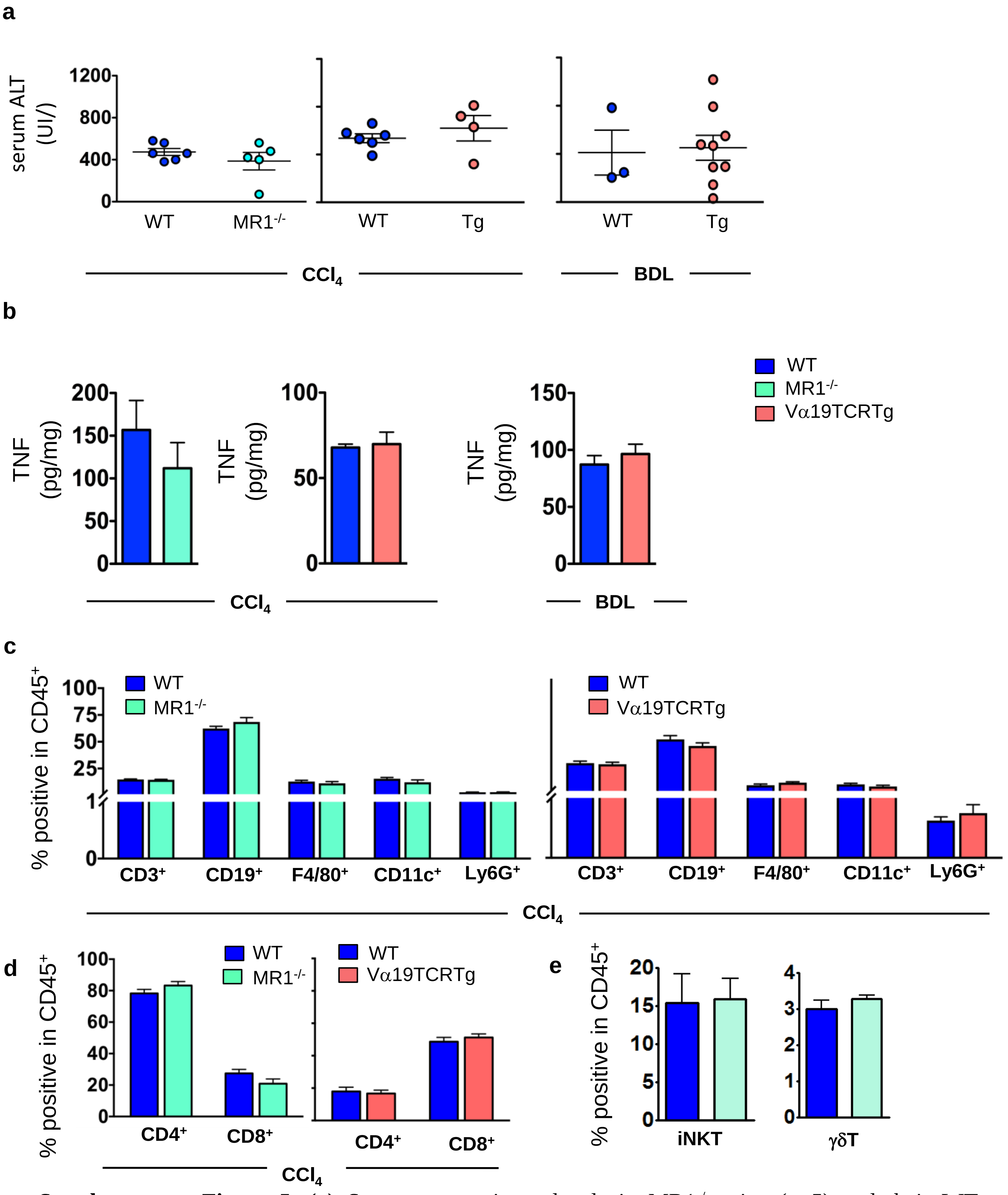

Supplementary Figure 5. (a) Serum transaminase levels in MR1$^{-/-}$ mice (n=5) and their WT littermates (n=6) chronically administered CCl_4**;** Vα19TCRTg mice (n=4) and their WT littermates (n=5) chronically exposed to CCl_4 or Vα19TCRTg mice (n=9) and their WT littermates (n=3) subjected to bile-duct ligation. **(b)** Hepatic TNF levels in MR1$^{-/-}$ mice (n=5) and their WT littermates (n=6) **;** Vα19TCRTg mice (n=5) and their WT littermates (n=4) chronically administered CCl_4; Vα19TCRTg mice (n=9) and their WT littermates (n=3) subjected to bile-duct ligation. **(c)** and **(d)** Comparison of intrahepatic immune cell subsets and T lymphocyte populations (CD4 and CD8) in MR1$^{-/-}$ mice (n=5) and their WT littermates (n=6), Vα19TCRTg mice (n=4) and their WT littermates (n=5) chronically exposed to CCl_4. (e) Frequency of iNKT and γδT cells among CD45 cells in the liver from MR1$^{-/-}$ mice and their WT littermates (n=5 WT, n=3 KO for iNKT and n=9 WT, n=3 KO for γδT. Statistical analysis was performed using Mann-Whitney test (a-e) .

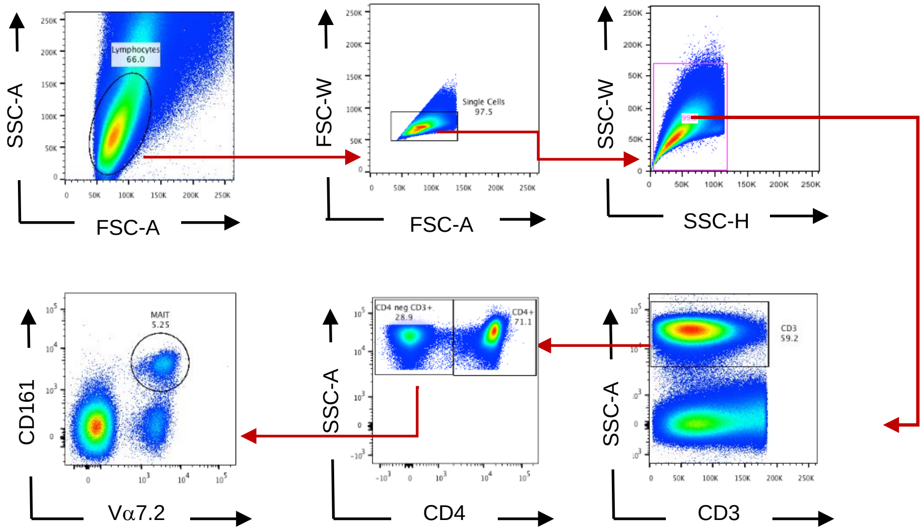

Supplementary Figure 6. Gating strategy of MAIT cells in human blood. Representative dot plots describing the analysis of frequency of blood MAIT cells among five millions of PBMC from a healthy donor. MAIT cells are defined as CD3^{+}CD4^{-}CD161^{+}Vα7.2^{+} cells in the lymphocyte gate.

Supplementary Table 1: Characteristics of healthy donors and cirrhotic patients used for studies in blood samples.

Characteristics	Controls	Alcoholic cirrhosis	NASH-related cirrhosis
n	47	63	11
Age (years)	34 (29-53)	57 (50-63)	60 (57-65)
Male gender (%)	23 (48.9)	52 (83)	9 (82)
Hb (g/dl)		10.6 (9.3-12.9)	12.8 (11.5-14.6)
Platelets (G/l)		115 (73-162)	92 (72-233)
Leukocytes (G/l)		6.2 (4.5-9.3)	6.5 (3.2-7.6)
Lymphocytes (G/l)		1.3 (0.8-1.8)	1.9 (0.7-2.4)
Neutrophils (G/l)		2.2 (3.8-6.2)	3.8 (2.4-5.0)
Monocytes (G/l)		0.8 (0.4-1.0)	0.5 (0.4-0.7)
Serum Sodium (mmol/l)		135 (132-136)	137 (143-139)
Serum Bilirubin (mg/dl)		3.0 (1.2-7.7)	1.3 (0.8-2.1)
Serum Creatinine (mg/dl)		0.7 (0.6-0.9)	0.8 (0.7-0.9)
Serum Albumin (g/l)		28 (22-33)	34.5 (28.8-41.2)
INR		1.5 (1.2-1.9)	1.2 (1.0-1.4)
SGOT (IU/l)		61 (41-83)	44 (30-59)
SGPT (IU/l)		33 (21-54)	37 (33-60)
Ascites (%)		48 (76)	2 (18)
Encephalopathy (%)		13 (21)	1 (9)
Compensated cirrhosis(%)		9 (14)	6 (55)
Decompensated cirrhosis (%)		54 (86)	5 (45)
Child-Pugh Score		10 (8-12)	6 (5-9)
MELD score		14.8 (10.3-20.3)	11.5 (7.3-15.2)
Beta-Blockers (%)		21 (33.3)	7 (63.6)
Chronic antibiotic prophylaxis (Noroxin and/or Rifaximin) (%)		8 (13)	2 (18)
Corticosteroids (%)		1 (1.6)	0 (0)

Results are expressed as number (percentage) or as median (IQR)

Supplementary Table 2: Analysis of Vα7.2 staining localization in human liver from control (F0), patients with mild fibrosis (F1) and cirrhotic patients (F4).

	Liver sample number	Gender	Age	Cirrhosis etiology	Liver sample source	Fibrosis stage	Vα7.2 in sinusoidal space	Vα7.2 in mesenchymal space with or without fibrosis
CONTROL LIVERS	**1**	Female	61	–	Colorectal cancer metastasis	F0	+	0
	**2**	Male	65	–	Kidney cancer metastasis	F0	+	0
	**3**	Male	56	–	Non hepatocellular primary tumor	F0	+	0
	**4**	Female	60	–	Non hepatocellular primary tumor	F0	+	0
	**5**	Female	75	–	Colorectal cancer metastasis	F1	+	0
	**6**	Male	53	–	Non hepatocellular primary tumor	F1	+	0
	**7**	Male	77	–	Hepatocellular carcinoma	F1	+	+
CIRRHOTIC LIVERS	**8**	Male	57	Alcohol and metabolic syndrome	Liver explant	F4	0	+
	**9**	Female	68	Metabolic syndrome	Hepatocellular carcinoma	F4	0	+
	**10**	Male	61	Hepatitis C	Hepatocellular carcinoma	F3-F4	+	+
	**11**	Male	53	Metabolic syndrome	Liver explant	F4	+	+
	**12**	Male	56	Alcohol	Liver explant	F4	0	+

n=7 for control (F0 and F1) livers, n=5 for cirrhotic livers(F3/F4). Staining: 0 = negative, + = positive. p=0.015 for control vs cirrhotic Vα7.2 staining in mesenchymal space and p=0.045 for Vα7.2 staining in sinusoidal space by Fisher's test.
